# Supplementary material for: Senescent cells perturb intestinal stem cell differentiation through Ptk7 induced noncanonical Wnt and YAP signaling
Source: Nat Commun. 2023 Jan 11;14:156. doi: 10.1038/s41467-022-35487-9 (PMC9834240; doi:10.1038/s41467-022-35487-9)
Supplement: Supplementary file 1 — Supplementary Information [file 41467_2022_35487_MOESM1_ESM.pdf]

Yun et al.: Senescent cells perturb intestinal stem cell differentiation through Ptk7 induced noncanonical Wnt and YAP signaling

Supplementary Materials

Supplementary Figure 1

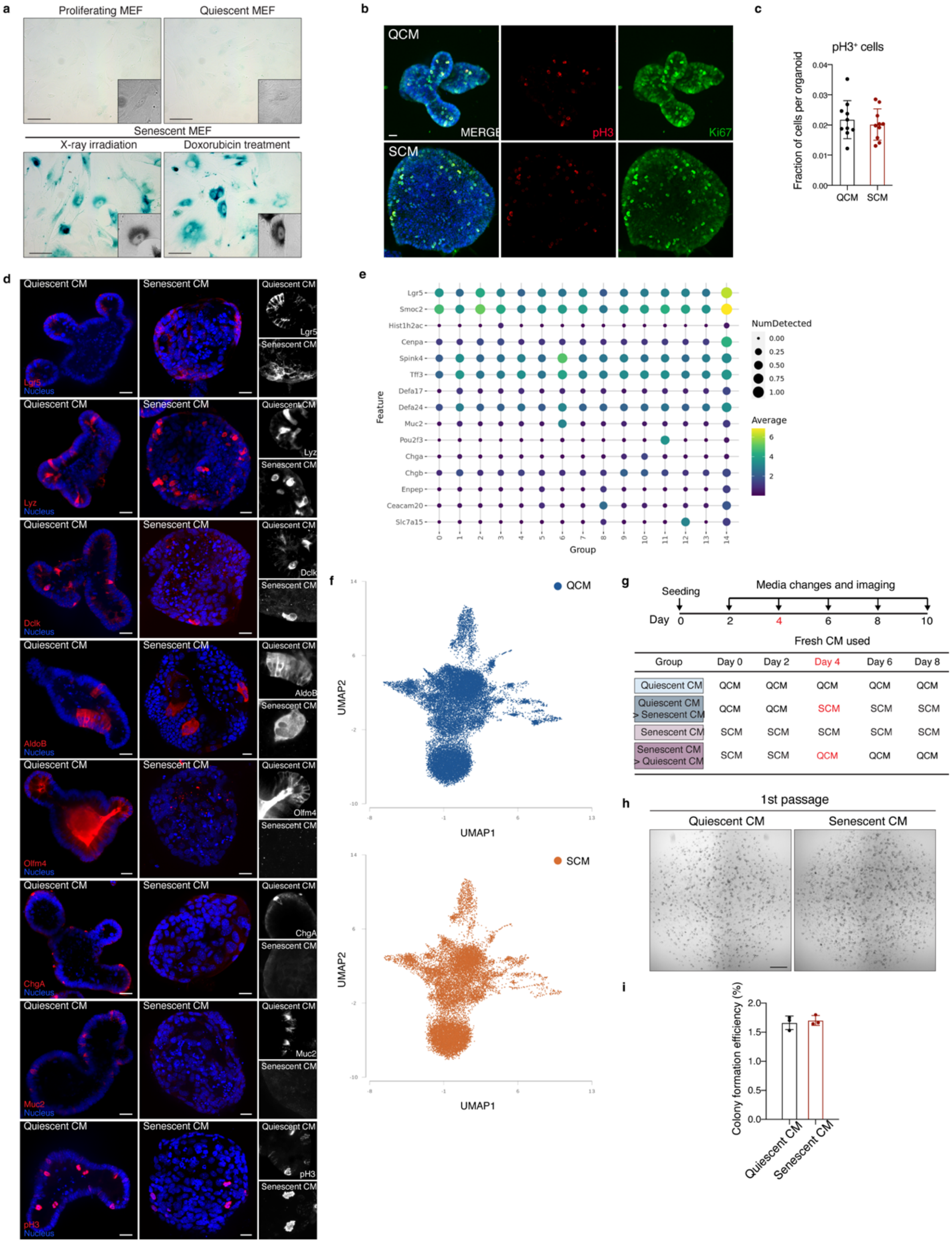

Supplementary Figure 1 (corresponding to Fig. 1).

(a) Representative bright field images of SA-β-gal stain in proliferating, quiescent, and senescent MEFs. Inset images show enlarged black and white images of boxed area. Scale bar: 100 μm. (b) Whole mount immunostaining of organoids for pH3 and Ki67. pH3, Ki67, and nucleus are shown in red, green, and blue respectively. Scale bar: 20 μm. (c) Fraction of phospho-Histone H3 positive cells in organoids exposed to QCM or SCM. Data are presented as the mean ± SD. n=10. (d) Whole mount immunostaining of organoids for various cell type markers. Cell type markers and nucleus are shown in red and blue respectively. Black and while enlarged images are shown at the right. Scale bar: 20 μm. n=10. (e) Identification of marker genes for each cluster, with the size of the dot corresponding to the percentage of nuclei within the nuclei expressing the gene. (f) UMAP projections showing contribution of nuclei from QCM and SCM exposed organoids. QCM was labeled in blue (top), and SCM was labeled in orange (bottom). (g) Schematic diagram showing the timeline of CM changes and imaging for each condition. (h) Representative images of the colony formation efficiency assay of organoids passaged after exposed to quiescent or senescent conditioned media and cultured in normal organoid culture media. Scale bar: 500 µm. (i) Quantification of data from (h). The percentage of cells that form organoids over a total number of seeded cells is shown (mean ± s. d.; two-tailed t-test).

Supplementary Figure 2

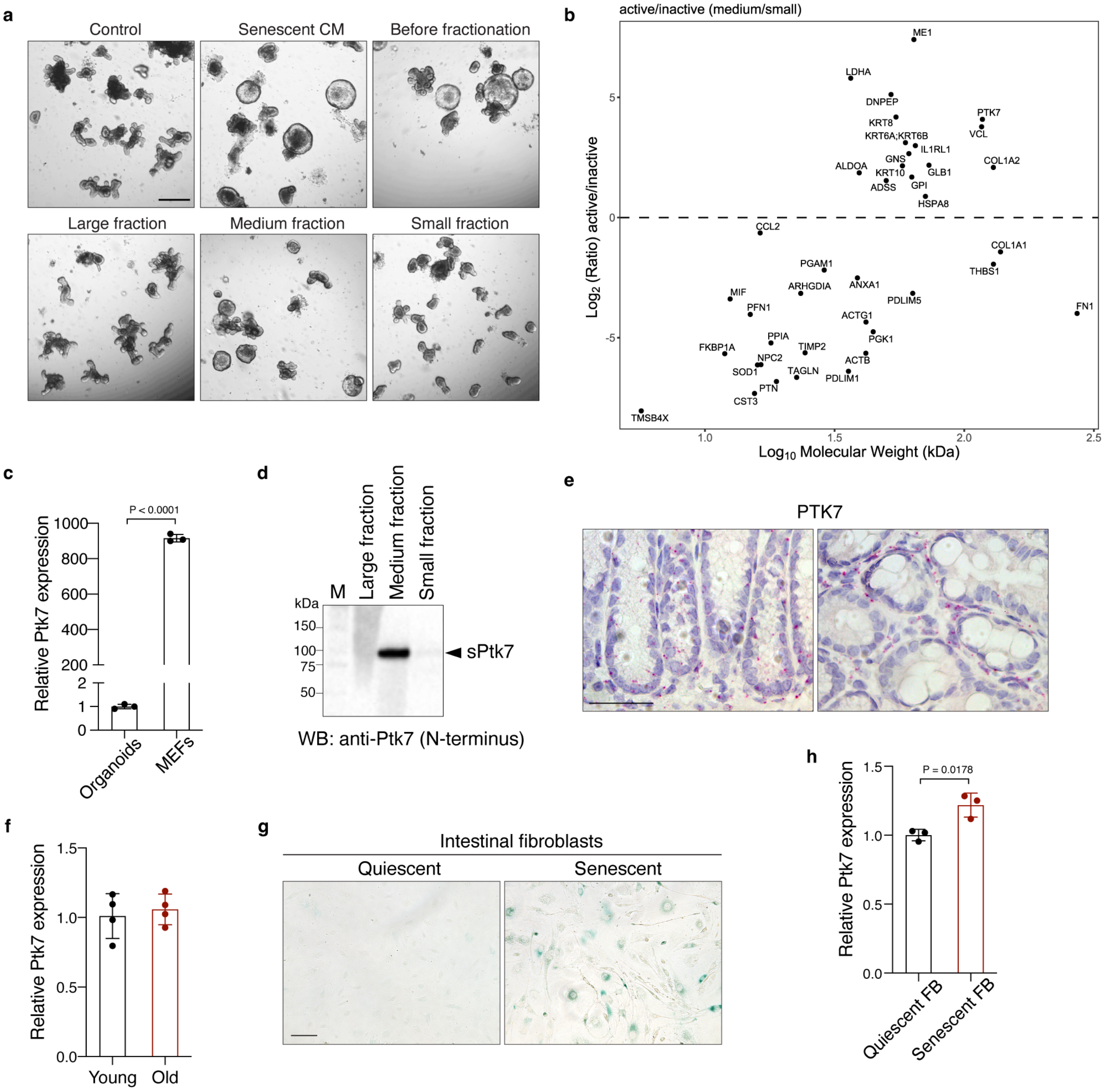

Supplementary Figure 2 (corresponding to Fig. 2 and 3).

(a) Representative images of organoids cultured in different fractions of senescent conditioned media collected from size exchange column. Scale bar, 200 µm. (b) Scatterplot of active:nonactive ratio (log2) versus molecular weight (log10). (c) The relative *PTK7* expression in intestinal organoids and MEFs (mean ± s. d.; n=3; two-tailed t-test). (d) Western blot for sPtk7 in different fractions of senescent conditioned media. An arrow head indicates soluble form of Ptk7; sPtk7. (e) Ptk7 transcripts in the mouse large intestine detected by RNAscope in situ hybridization. Longitudinal (left) and cross (right) sections were shown. Scale bar: 50 µm. (f) The

relative *PTK7* expression in the intestine from young and old mice (mean ± s. d.; n=4; two-tailed t-test). (g) Representative bright field images of SA-β-gal stain quiescent and senescent primary intestinal fibroblasts. Scale bar: 100 μm. (h) *PTK7* expression in quiescent and senescent intestinal fibroblasts as a ratio relative to quiescent intestinal fibroblasts. (mean ± s. d.; n=3; two-tailed t-test).

Supplementary Figure 3

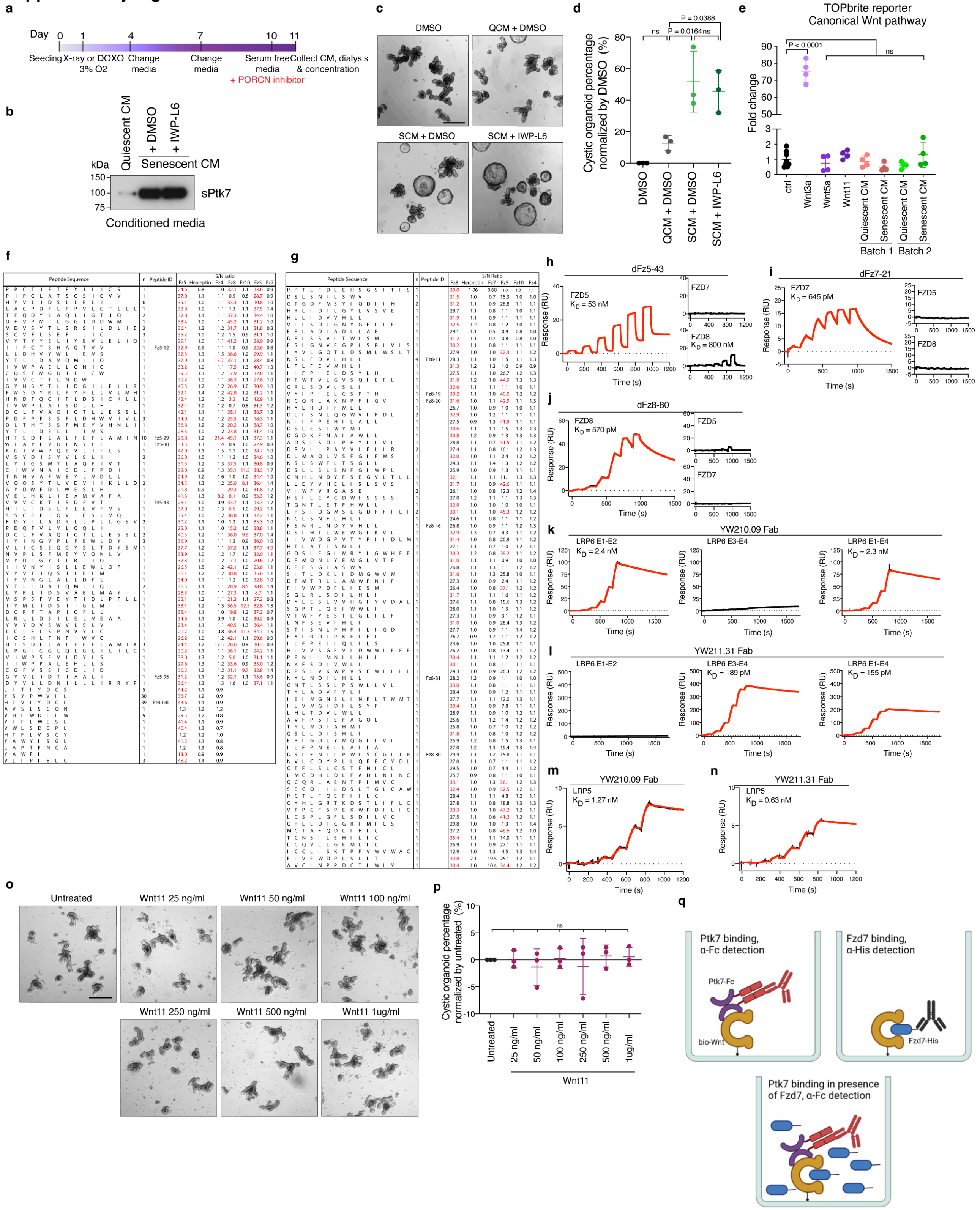

Supplementary Figure 3 (corresponding to Fig. 4).

(a) Schematic diagram showing induction of cellular senescence in fibroblasts, PORCN inhibitor treatment, and conditioned media collection. (b) Western blot for sPtk7 in CM collected from quiescent and senescent MEFs treated with vehicle or PORCN inhibitor for 24 hrs. Conditioned media corresponding to the same number of cells was loaded. (c) Representative images of organoids cultured in corresponding CM. Scale bar, 200 μm. (d) Quantification of data from (c) shows no change in the percentage of cystic organoids by inhibition of Wnt secretion. Data are presented as means ± s. d. One-way ANOVA followed by Tukey's post-hoc multiple comparison tests. Each dot represents a result from organoids established from a mouse, n=3. (e) TOPbrite dual-luciferase Wnt reporter assay. The graph shows fold changes in luminescence for the indicated treatments normalized to control. Data are presented as means ± s. d. One-way ANOVA followed by Tukey's post-hoc multiple comparison tests. n=4.

(f) Sequences and binding of phage display derived peptides selected against hFZD5-CRD. Peptide sequences are given in single letter amino acid code. n reflects how often the peptide sequence was found in the analyzed phage pool. Peptide ID shows the name of peptides that were synthesized and further analyzed. S/N ratio gives the ratio of phage ELISA signal with immobilized FZD-CRDs or Herceptin over signal with no immobilized protein (BSA blocking only). **(g)** Sequences and binding of phage display derived peptides selected against hFZD8-CRD. Peptide sequences are given in single letter amino acid code. n reflects how often the peptide sequence was found in the analyzed phage pool. Peptide ID shows the name of peptides that were synthesized and further analyzed. S/N ratio gives the ratio of phage ELISA signal with immobilized FZD-CRDs or Herceptin over signal with no immobilized protein (BSA blocking only).

(h-j) Surface plasmon resonance (SPR) of dFz5-43, dFz7-21, and dFz8-80. (h) Binding of peptide dFz5-43 to different human FZD-CDRs probed by surface plasmon resonance. Binding response is shown in black. For FZD5-CDR, the fit to a 1:1 kinetic model is shown in red. Equilibrium fitting with resulting KDs is shown. (i) Binding of peptide dFz7-21 to different human FZD-CDRs probed by surface plasmon resonance. Binding response is shown in black. For FZD7-CDR, the fit to a 1:1 kinetic model is shown in red. Equilibrium fitting with resulting KDs is shown. (j) Binding of peptide dFz8-80 to different human FZD-CDRs probed by surface plasmon resonance. Binding response is shown in black. For FZD8-CDR, the fit to a 1:1 kinetic model is shown in red. Equilibrium fitting with resulting KDs is shown. (k-n) Surface plasmon resonance (SPR) of YW210.09 and YW211.31.62. Binding of Fabs YW210.09 and YW211.31.62 to the extracellular domain of LRP6 (k, l) and to the extracellular domain of LRP5 (m, n). Binding responses are shown in black overlaid with fit to a 1:1 kinetic model in red. (o) Representative images of

organoids treated with different concentrations of recombinant Wnt11. Scale bar, 200 μm. (p) Quantification of data from (o) shows no effect on intestinal organoid morphology by Wnt11. Data are presented as means ± s. d. One-way ANOVA followed by Tukey's post-hoc multiple comparison tests. Each dot represents a result from organoids established from a mouse, n=3. (q) Schematic diagram illustrating interaction of Ptk7, Wnt ligands, and Fzd7. The figure was created with BioRender.com.

Supplementary Figure 4

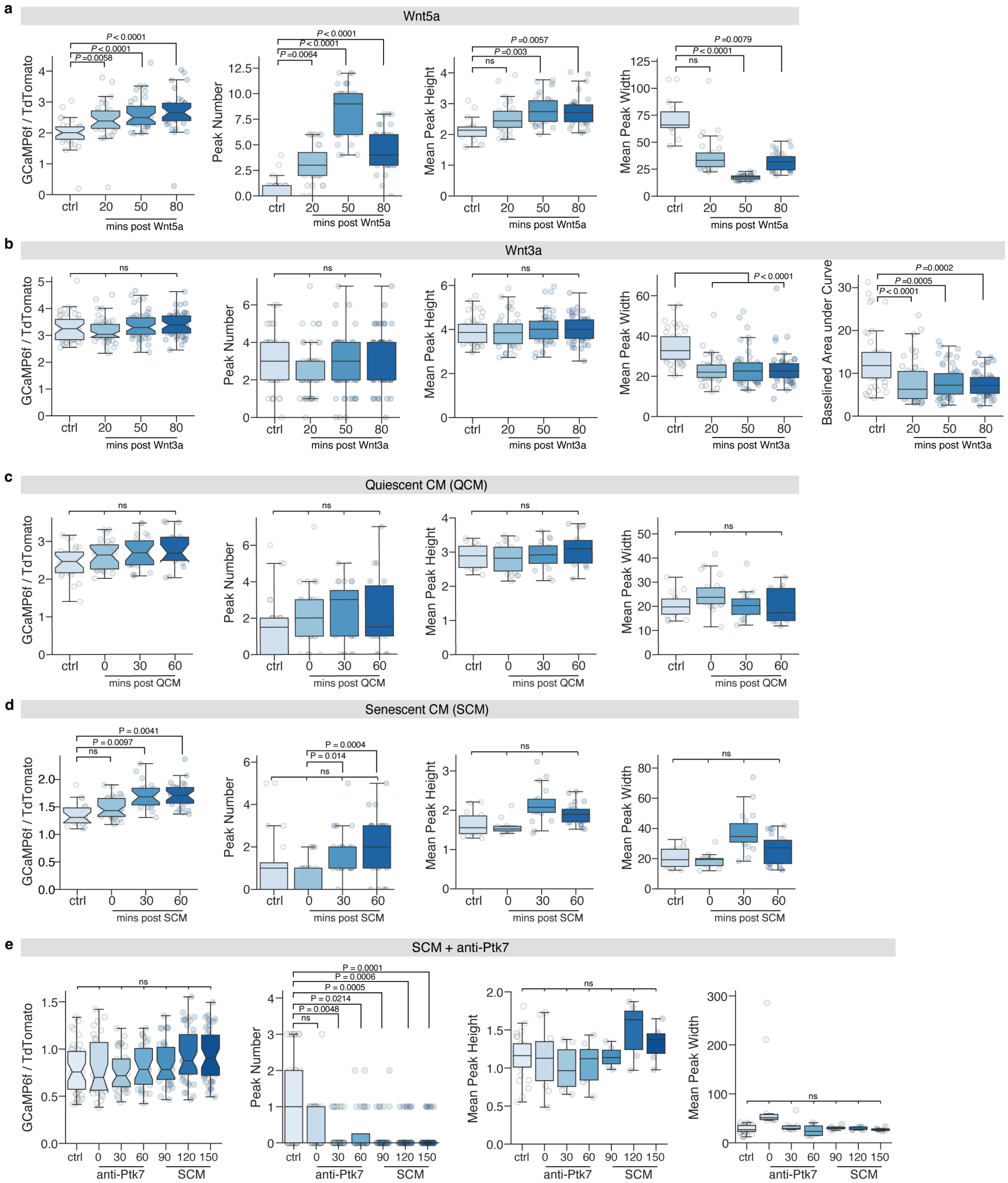

Supplementary Figure 4 (corresponding to Fig. 5).

(a-e) Boxplots showing GCaMP6f/TdTomato intensity ratio, Ca^{2+} peak number, mean Ca^{2+} peak height, and mean Ca^{2+} peak width of each time points from live imaging before and after addition of Wnt5a (a), Wnt3a (b), quiescent CM (c), senescent CM (d), and anti-Ptk7 antibodies followed by senescent CM (e). Boxplots display median ± interquartile range with whiskers marking 1.5 times the interquartile range. The graph shows results from a representative experiment. Kruskal-Wallis test followed by Dunn's post-hoc multiple comparison tests. n=5, 5, 3, 6, and 6 in (a), (b), (c), (d), and (e) respectively.

Supplementary Figure 5

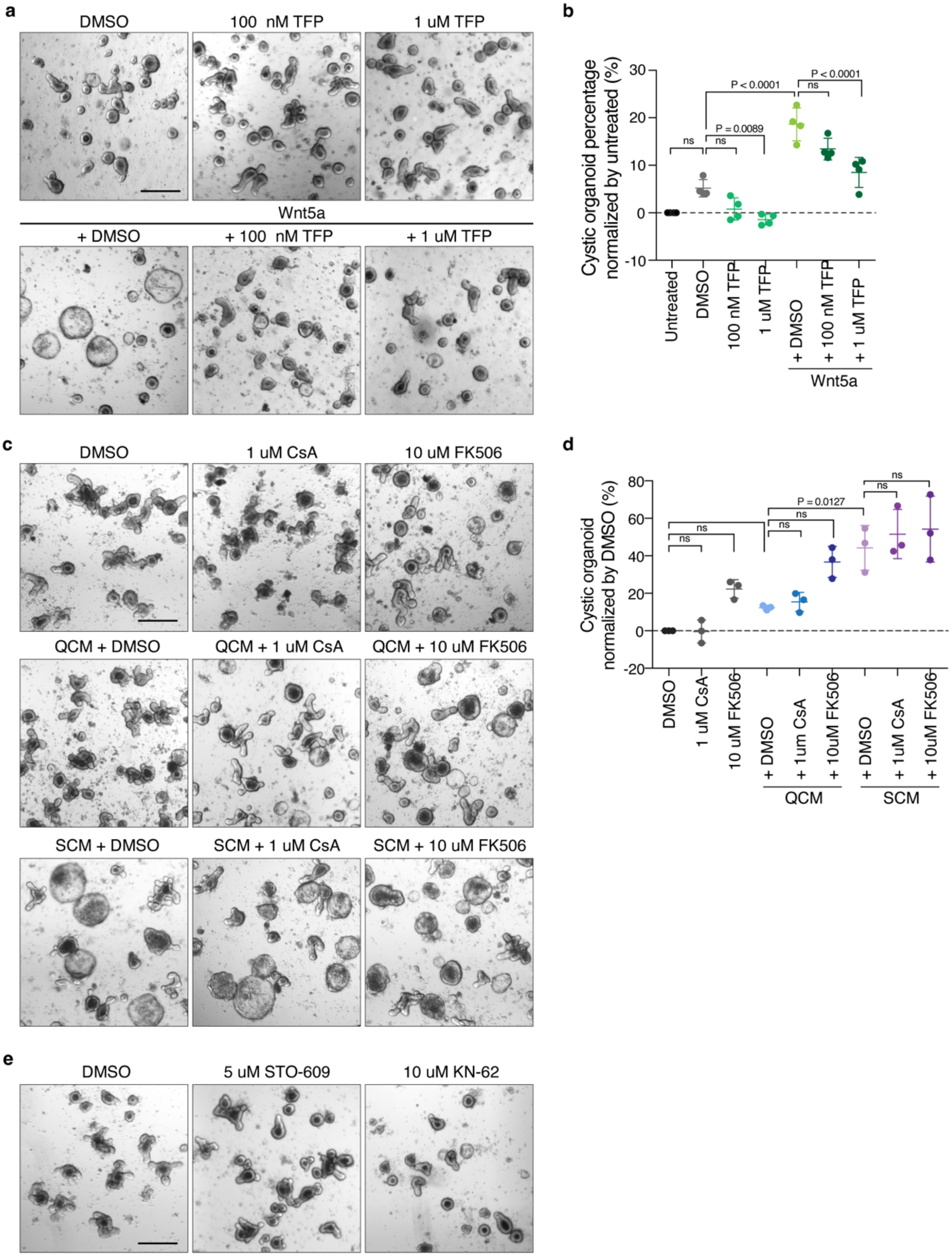

Supplementary Figure 5 (corresponding to Fig. 5).

(a) Representative images of organoids cultured in Wnt5a with DMSO, 100 nM TFP, or 1 μM TFP. Scale bar: 200 μm. (b) Quantification of data from (a). (c) Representative images of organoids cultured in QCM or SCM with DMSO, 1 μM CsA, or 10 μM FK506. Scale bar: 200 μm. (d) Quantification of data from (c). In (b) and (d), data are presented as means ± s. d. percentage of cystic organoids. Each dot represents a result from organoids established from a mouse, n=4 and 3 in (b) and (d) respectively. One-way ANOVA followed by Tukey's post-hoc multiple comparison tests. (e) Representative images of organoids treated with DMSO, 5 μM STO-609, or 10 μM KN-62. Scale bar: 200 μm

Supplementary Figure 6

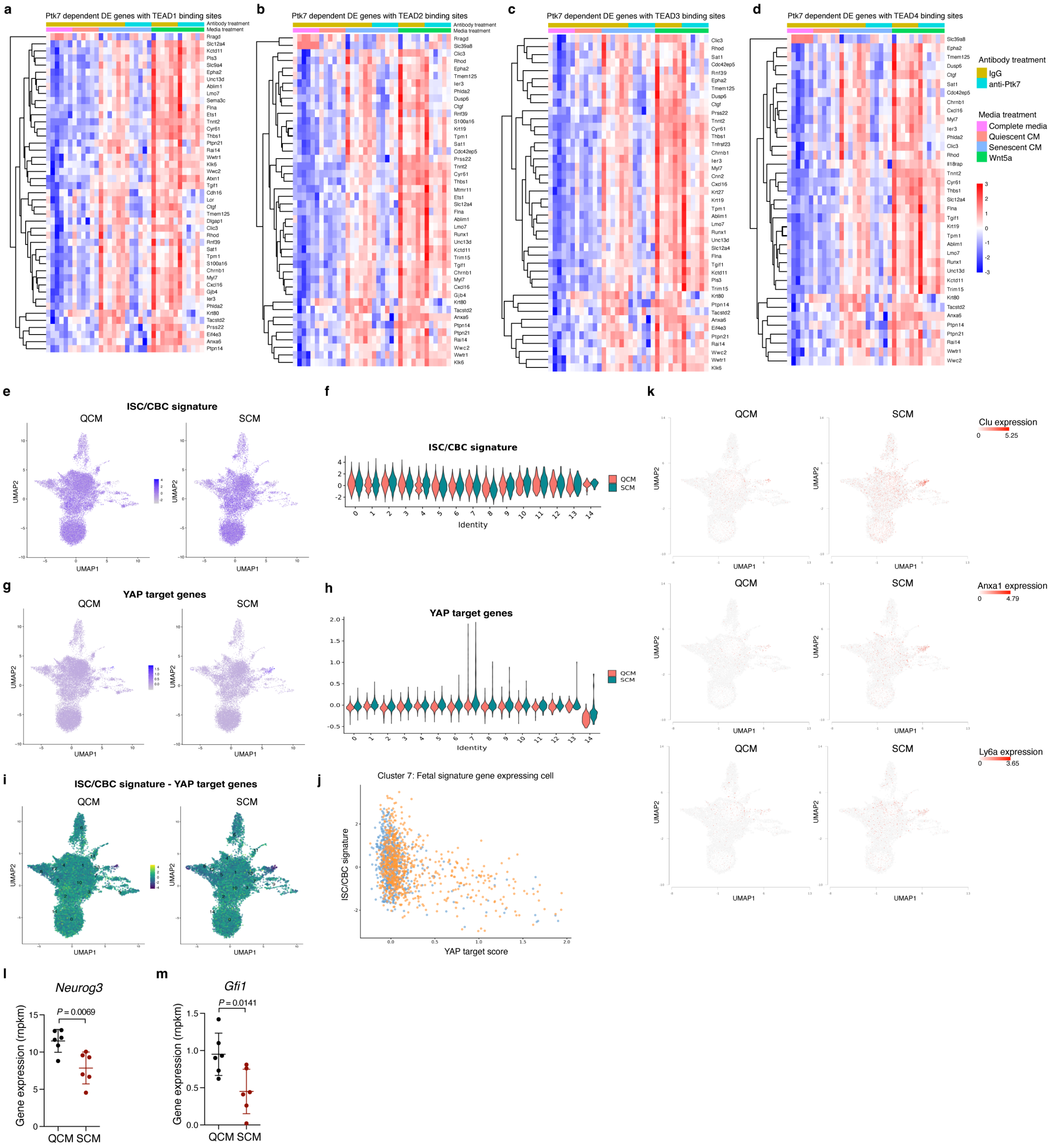

Supplementary Figure 6 (corresponding to Fig. 6).

(a-d) Heatmaps showing expression of Ptk7 dependent DE genes with different TEAD binding motifs- TEAD1 (a), TEAD2 (b), TEAD3 (c), and TEAD4 (d). (e-h) UMAPs and violin plots for the relative mean expression levels of ISC/CBC signature genes (e, f) and YAP target genes (g, h). P-values for each cluster in (h) - cluster 0 is p= 0.00046, clusters 2, 5, 6, 7, 8, 9, 14 are p<0.0001, cluster 3 is p=0.005, and cluster 10 is p=0.038. Two-tailed t-test. (i) Difference in ISC/CBC signature and YAP target genes on the UMAPs. (j) Cluster 7 fetal signature gene expressing cells plotted for ISC/CBC signature and YAP target score. (k) Expression of fetal signature genes; Clu (top), Anxa1 (middle), and Ly6a (bottom) on the UMAP. (l, m) *Neurog3* and *Gfi1* expression in organoids exposed to QCM and SCM. mean ± s. d.; n=6; two-tailed t-test.

Supplementary Figure 7

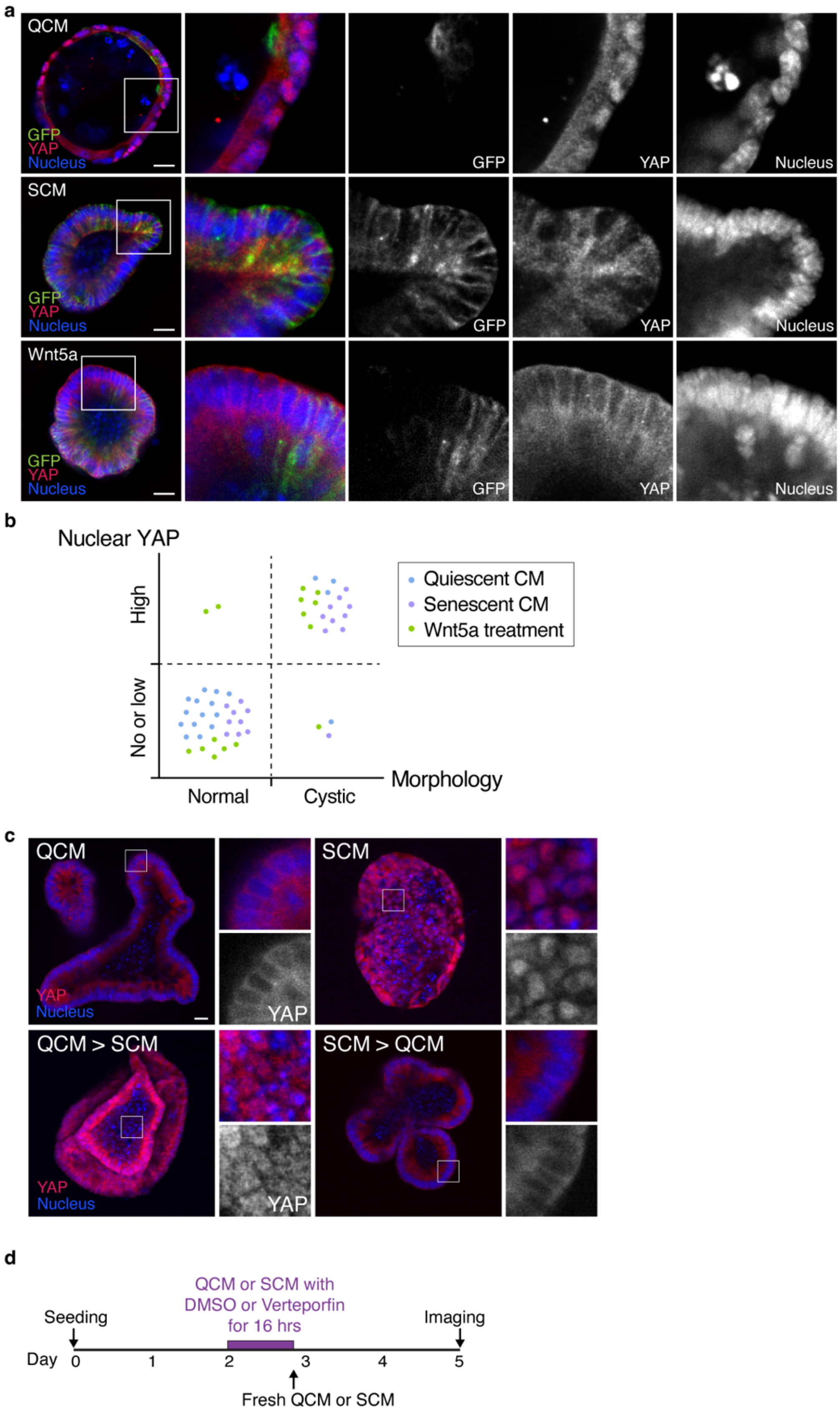

Supplementary Figure 7 (corresponding to Fig. 7).

(a) Whole mount immunostaining of organoids for YAP. Organoids established from Lgr5-DTR-GFP mice were stained for GFP, YAP, and nucleus (DAPI) in green, red, and blue respectively. Nuclear localization of YAP is observed in cystic organoids grown in quiescent conditioned media. Enlarged images of boxed area are shown at the right. Scale bar: 20 μm. (b) Correlation between nuclear YAP levels and organoid morphology. Each dot represents an organoid from QCM, SCM, and Wnt5a labeled in blue, purple, and green respectively. (c) Whole mount immunostaining of organoids for YAP. YAP and nucleus are shown in red and blue respectively. Representative images of an organoid from each condition are shown. n=4 per each condition. Enlarged images of boxed area are shown at the right. Scale bar: 20 μm. (d) Schematic diagram showing changes of CM and addition of DMSO or Verteporfin.

Supplementary Table 1: List of TaqMan assay probes used for qPCR

Target	Company	Cat. No.
GAPDH	Thermo Fisher Scientific	Mm99999915_g1
PTK7	Thermo Fisher Scientific	Mm00613367_g1
LGR5	Thermo Fisher Scientific	Mm00438890_m1
OLFM4	Thermo Fisher Scientific	Mm01320260_m1
Lyz1	Thermo Fisher Scientific	Mm00657323_m1
CHGA	Thermo Fisher Scientific	Mm00514341_m1
MUC2	Thermo Fisher Scientific	Mm00458299_m1
ALPI	Thermo Fisher Scientific	Mm01285814_g1
MKI67	Thermo Fisher Scientific	Mm01278617_m1

Supplementary Table 2: Antibodies used in this study

Antibody	Company	Cat. No.	Dilution
anti-Ki67	BD Biosciences	550609	1:100
anti-phospho-Histone H3	EMD Millipore	06-570	1:250
anti-GFP	Abcam	ab13970	1:400
anti-Olfm4	Cell Signaling Technology	39141S	1:200
anti-Lyz	Dako	EC 3.2.1.17	1:200
anti-ChgA	Novus Biologicals Biologicals	NB120-15160	1:200
anti-Dclk	Abcam	ab31704	1:100
anti-Muc2	Abcam	ab134119	1:100
anti-Aldolase B	Abcam	ab75751	1:100
Anti-Lrp5/6 Fabs	-	-	10 μg/ml
Rabbit IgG	Thermo Fisher Scientific	02-6102	5 μg/ml
anti-Ptk7	Sigma-Aldrich	HPA003222	5 μg/ml or 1:500
anti-YAP	Cell Signaling Technology	14074S	1:200
anti-MMP14	Abcam	ab51074	1:2000
anti-alpha-Tubulin	Cell Signaling Technology	3873S	1:5000
anti-beta-Actin	Cell Signaling Technology	8457S	1:10000
anti-Vimentin	Sigma-Aldrich	AB5733	1:200
anti-hIgG1-Fc-HRP	Thermo Fisher Scientific	A-10648	1:5000
anti-His-tag-HRP	Abcam	ab1187	1:5000
anti-rabbit IgG-HRP	GE Healthcare	NA934	1:5000
anti-chicken IgY Alexa Fluor 488	Jackson ImmunoResearch Laboratories	703-545-155	1:500
Anti-rabbit IgG Alexa Fluor Plus 555	Thermo Fisher Scientific	A32794	1:500

Supplementary Table 3: Recombinant proteins used in this study

Recombinant protein	Company	Cat. No.
hmWnt5a	R&D Systems	645-WN-010
Biotinylated hmWnt5a	R&D Systems	BT645
mWnt3a	R&D Systems	1324-WN-010
Biotinylated mWnt3a	R&D Systems	BT1324
hWnt11	R&D Systems	6179-WN-010
Ptk7	R&D Systems	9799-TK
FZD5	R&D Systems	5988-FZ
FZD7	R&D Systems	6178-FZ
FZD8	R&D Systems	6129-FZ
LRP5	R&D Systems	7344-LR

Supplementary Table 4: Compounds and inhibitors used in this study

Compound	Company	Cat. No.	Concentration
Doxorubicin	Sigma-Aldrich	44583	250 nM
DMSO	Sigma-Aldrich	D2650	-
TAPI-1	Sigma-Aldrich	579053	50 uM
GM6001	Sigma-Aldrich	364206	20 uM
IWP-L6	Sigma-Aldrich	5048190001	40 nM
Trifluoperazine	Sigma-Aldrich	T8516	1 uM
STO-609	Selleck Chemicals	S8274	5 uM
KN-62	Selleck Chemicals	S7422	10 uM
Cyclosporin A	Selleck Chemicals	S2286	1 uM
FK506	Selleck Chemicals	S5003	10 uM
Verteporfin	Sigma-Aldrich	SML0534	250 nM, 500 nM
TED347	Selleck Chemicals	S8951	2 uM
dFz5-43	-	-	80 uM
dFz7-21	-	-	50 uM
dFz8-80	-	-	80 uM
