# Supplementary material for: Senescent cells perturb intestinal stem cell differentiation through Ptk7 induced noncanonical Wnt and YAP signaling
Source: Nat Commun. 2023 Jan 11;14:156. doi: 10.1038/s41467-022-35487-9 (PMC9834240; doi:10.1038/s41467-022-35487-9)
Supplement: Supplementary file 3 — Description of Additional Supplementary Files [file 41467_2022_35487_MOESM3_ESM.pdf]

Description of Additional Supplementary Files

File Name: Supplementary Data 1
Description: All quantified proteins from data-independent acquisitions (DIA): active (medium) and non-active (small) fractions

File Name: Supplementary Data 2
Description: List of 39 proteins that are abundant in medium (active) and small (non-active) fractions

File Name: Supplementary Data 3
Description: List of proteins from this study that overlap with SASP factors identified in the SASP Altas

File Name: Supplementary Data 4
Description: Ptk7 peptide sequences detected from MS

File Name: Supplementary Movie 1
Description: Ca^{2+} oscillations in the crypt domain of a mouse intestinal organoid. GCaMP6f in green and tdTomato in red.

File Name: Supplementary Movie 2
Description: Baseline Ca^{2+} oscillations in an intestinal organoid before Wnt5a treatment. GCaMP6f in green and tdTomato in red.

File Name: Supplementary Movie 3
Description: Ca^{2+} oscillations in an intestinal organoid 50 min after Wnt5a treatment. GCaMP6f in green and tdTomato in red.
