# Supplementary material for: Senescent cells perturb intestinal stem cell differentiation through Ptk7 induced noncanonical Wnt and YAP signaling
Source: Nat Commun. 2023 Jan 11;14:156. doi: 10.1038/s41467-022-35487-9 (PMC9834240; doi:10.1038/s41467-022-35487-9)
Supplement: Supplementary file 8 — Reporting Summary [file 41467_2022_35487_MOESM8_ESM.pdf]

nature portfolio

Corresponding author(s): Rami N. Hannoush and Heinrich Jasper

Last updated by author(s): Nov 8, 2022

Reporting Summary

Nature Portfolio wishes to improve the reproducibility of the work that we publish. This form provides structure for consistency and transparency in reporting. For further information on Nature Portfolio policies, see our Editorial Policies and the Editorial Policy Checklist.

Statistics

For all statistical analyses, confirm that the following items are present in the figure legend, table legend, main text, or Methods section.

n/a	Confirmed	
☐	☒	The exact sample size (*n*) for each experimental group/condition, given as a discrete number and unit of measurement
☐	☒	A statement on whether measurements were taken from distinct samples or whether the same sample was measured repeatedly
☐	☒	The statistical test(s) used AND whether they are one- or two-sided<br>*Only common tests should be described solely by name; describe more complex techniques in the Methods section.*
☒	☐	A description of all covariates tested
☐	☒	A description of any assumptions or corrections, such as tests of normality and adjustment for multiple comparisons
☐	☒	A full description of the statistical parameters including central tendency (e.g. means) or other basic estimates (e.g. regression coefficient) AND variation (e.g. standard deviation) or associated estimates of uncertainty (e.g. confidence intervals)
☐	☒	For null hypothesis testing, the test statistic (e.g. *F*, *t*, *r*) with confidence intervals, effect sizes, degrees of freedom and *P* value noted<br>*Give P values as exact values whenever suitable.*
☒	☐	For Bayesian analysis, information on the choice of priors and Markov chain Monte Carlo settings
☒	☐	For hierarchical and complex designs, identification of the appropriate level for tests and full reporting of outcomes
☒	☐	Estimates of effect sizes (e.g. Cohen's *d*, Pearson's *r*), indicating how they were calculated

Our web collection on statistics for biologists contains articles on many of the points above.

Software and code

Policy information about availability of computer code

Data collection	HTSeqGenie v4.2.2. GSNAP v2013-11-01; Paragon algorithm (4.5.0.0,1654);10x CellRanger
Data analysis	GraphPad Prism v7.02; the voom and the Limma R packages; RcisTarget; scikit-image; MATLAB; Scanpy; Harmony Clusters; Seurat; Python matplotlib; scran, seaborn, SciPy and scikit-posthoc packages.

For manuscripts utilizing custom algorithms or software that are central to the research but not yet described in published literature, software must be made available to editors and reviewers. We strongly encourage code deposition in a community repository (e.g. GitHub). See the Nature Portfolio guidelines for submitting code & software for further information.

Data

Policy information about availability of data

All manuscripts must include a data availability statement. This statement should provide the following information, where applicable:

- Accession codes, unique identifiers, or web links for publicly available datasets
- A description of any restrictions on data availability
- For clinical datasets or third party data, please ensure that the statement adheres to our policy

The mass spectrometric raw data are deposited at ftp://massive.ucsd.edu/MSV000088032/ with the MassIVE ID MSV000088032; it is also available at

ProteomeXchange with the ID PXD028176. RNAseq and snRNA-seq data will be deposited in the National Center for Biotechnology Information's (NCBI) GEO database.

Field-specific reporting

Please select the one below that is the best fit for your research. If you are not sure, read the appropriate sections before making your selection.

☒ Life sciences ☐ Behavioural & social sciences ☐ Ecological, evolutionary & environmental sciences

For a reference copy of the document with all sections, see nature.com/documents/nr-reporting-summary-flat.pdf

Life sciences study design

All studies must disclose on these points even when the disclosure is negative.

Sample size	No statistical methods were used to calculate the sample size. Sample sizes (numbers of independently derived organoid cultures) were determined based on variation observed in pilot studies and exact numbers are described in Figure legends.
Data exclusions	No data were excluded from analyses.
Replication	The number of replications for each experiment is listed in figure legends and materials. All attempts for replication were successful. MS screen and RNAseq screens were performed once for data gathering / hypothesis generation, and the data generated were validated by other methods.
Randomization	Animals for organoid generation were randomly selected. Organoids established from each individual mouse were used for both control and experimental groups and treated randomly.
Blinding	Image analysis, quantification, and data analysis were performed on samples that were blinded to the experimenter.

Reporting for specific materials, systems and methods

We require information from authors about some types of materials, experimental systems and methods used in many studies. Here, indicate whether each material, system or method listed is relevant to your study. If you are not sure if a list item applies to your research, read the appropriate section before selecting a response.

Materials & experimental systems

n/a	Involved in the study	
☐	☒	Antibodies
☐	☒	Eukaryotic cell lines
☒	☐	Palaeontology and archaeology
☐	☒	Animals and other organisms
☒	☐	Human research participants
☒	☐	Clinical data
☒	☐	Dual use research of concern

Methods

n/a	Involved in the study	
☒	☐	ChIP-seq
☒	☐	Flow cytometry
☒	☐	MRI-based neuroimaging

Antibodies

Antibodies used

anti-Ki67 BD Biosciences 550609
anti-phospho-Histone H3 EMD Millipore 06-570
anti-GFP Abcam ab13970
anti-Olfm4 Cell Signaling Technology 39141S
anti-Lyz Dako EC 3.2.1.17
anti-ChgA Novus Biologicals Biologicals NB120-15160
anti-Dclk Abcam ab31704
anti-Muc2 Abcam ab134119
anti-Aldolase B Abcam ab75751
Rabbit IgG Thermo Fisher Scientific 02-6102
anti-Ptk7 Sigma-Aldrich HPA003222
anti-YAP Cell Signaling Technology 14074S
anti-hIgG1-Fc-HRP Thermo Fisher Scientific A-10648
anti-His-tag-HRP Abcam ab1187
anti-rabbit IgG-HRP GE Healthcare NA934
anti-chicken IgY Alexa Fluor 488 Jackson ImmunoResearch Laboratories 703-545-155
Anti-rabbit IgG Alexa Fluor Plus 555 Thermo Fisher Scientific A32794
anti-MMP14 Abcam ab51074
anti-alpha-Tubulin Cell Signaling Technology 3873S

anti-beta-Actin Cell Signaling Technology 8457S
anti-Vimentin Sigma-Aldrich AB5733

Validation

Validation of all commercial antibodies are available at the manufacturer's websites. The validation reference for each antibody is listed below:
1. anti-Ki67 BD Biosciences 550609. https://www.bdbiosciences.com/en-us/products/reagents/flow-cytometry-reagents/research-reagents/single-color-antibodies-ruo/purified-mouse-anti-ki-67.550609
2. anti-phospho-Histone H3 EMD Millipore 06-570. https://www.emdmillipore.com/US/en/product/Anti-phospho-Histone-H3-Ser10-Antibody-Mitosis-Marker,MM_NF-06-570
3. anti-GFP Abcam ab13970. https://www.abcam.com/gfp-antibody-ab13970.html
4. anti-Olfm4 Cell Signaling Technology 39141S. https://www.cellsignal.com/products/primary-antibodies/olfm4-d6y5a-xp-rabbit-mab-mouse-specific/39141
5.anti-Lyz Dako EC 3.2.1.17. https://www.agilent.com/en/product/immunohistochemistry/antibodies-controls/primary-antibodies/lysozyme-ec-3-2-1-17-(concentrate)-76124
6. anti-ChgA Novus Biologicals Biologicals NB120-15160. https://www.novusbio.com/products/chromogranin-a-antibody_nb120-15160
7. anti-Dclk Abcam ab31704. https://www.abcam.com/dcamkl1-antibody-ab31704.html
8. anti-Muc2 Abcam ab134119. https://www.abcam.com/muc2-antibody-epr6145-ab134119.html
9. anti-Aldolase B Abcam ab75751. https://www.abcam.com/aldolase-b--aldolase-c-antibody-epr3138y-ab75751.html
10.Rabbit IgG Thermo Fisher Scientific 02-6102.https://www.thermofisher.com/antibody/product/Rabbit-IgG-Isotype-Control/02-6102
11.anti-Ptk7 Sigma-Aldrich HPA003222.https://www.sigmaaldrich.com/US/en/product/sigma/hpa003222
12.anti-YAP Cell Signaling Technology 14074S. https://www.cellsignal.com/products/primary-antibodies/yap-d8h1x-xp-rabbit-mab/14074
13.anti-hIgG1-Fc-HRP Thermo Fisher Scientific A-10648. https://www.thermofisher.com/antibody/product/Mouse-anti-Human-IgG1-Fc-Secondary-Antibody-clone-HP6069-Monoclonal/A-10648
14.anti-His-tag-HRP Abcam ab1187. https://www.abcam.com/hrp-6x-his-tag-antibody-ab1187.html
15.anti-rabbit IgG-HRP GE Healthcare NA934. https://www.sigmaaldrich.com/US/en/product/sigma/gena9341ml
16.anti-chicken IgY Alexa Fluor 488 Jackson ImmunoResearch Laboratories 703-545-155. https://www.jacksonimmuno.com/catalog/products/703-545-155
17.Anti-rabbit IgG Alexa Fluor Plus 555 Thermo Fisher Scientific A32794. https://www.thermofisher.com/antibody/product/Donkey-anti-Rabbit-IgG-H-L-Highly-Cross-Adsorbed-Secondary-Antibody-Polyclonal/A32794
18. anti-MMP14 Abcam ab51074. https://www.abcam.com/mmp14-antibody-ep1264y-ab51074.html
19.anti-alpha-Tubulin Cell Signaling Technology 3873S. https://www.cellsignal.com/products/primary-antibodies/a-tubulin-dm1a-mouse-mab/3873
20.anti-beta-Actin Cell Signaling Technology 8457S. https://www.cellsignal.com/products/primary-antibodies/b-actin-d6a8-rabbit-mab/8457?site-search-type=Products&N=4294956287&Ntt=8457s&fromPage=plp&_requestid=2829368
21. anti-Vimentin Sigma-Aldrich AB5733. https://www.emdmillipore.com/US/en/product/Anti-Vimentin-Antibody,MM_NF-AB5733?ReferrerURL=https%3A%2F%2Fwww.google.com%2F

Eukaryotic cell lines

Policy information about cell lines

Cell line source(s)	HEK293-TB, sourced at Genentech was used for reporter assays
Authentication	HEK293-TB was generated, tested, and published previously (please see below), and authenticated here based on the responsiveness of the luciferase reporters.<br>Zhang et al., Nature Chemical Biology volume 5, pages217–219 (2009)<br>Nile et al, Nature Chemical Biology volume 14, pages582–590 (2018)
Mycoplasma contamination	The cell line was not tested for Mycoplasma contamination.
Commonly misidentified lines (See ICLAC register)	NA

Animals and other organisms

Policy information about studies involving animals; ARRIVE guidelines recommended for reporting animal research

Laboratory animals	All mice were housed in a Specific Pathogen Free (SPF) facility, a HEPA filtered room and using a Tecniplast individually ventilated caging system / room. The room has controlled temperature (20-22°C), humidity (30%–70%) and light (12 hour light-dark cycle).
Wild animals	No wild animals were used in the study.
Field-collected samples	No field collected samples were used in the study.
Ethics oversight	All mice were used in accordance with protocols approved by Genentech’s Institutional Animal Care and Use Committee and adhere to the NRC Guidelines for the Care and Use of Laboratory Animals.

Note that full information on the approval of the study protocol must also be provided in the manuscript.
